# Critical roles of lncRNA-mediated autophagy in urologic malignancies

**DOI:** 10.3389/fphar.2024.1405199

**Published:** 2024-06-13

**Authors:** Lifeng Gan, Liying Zheng, Junrong Zou, Peiyue Luo, Tao Chen, Jun Zou, Wei Li, Qi Chen, Le Cheng, Fangtao Zhang, Biao Qian

**Affiliations:** ^1^ The First Clinical College, Gannan Medical University, Ganzhou, Jiangxi, China; ^2^ Department of Urology, The First Affiliated Hospital of Gannan Medical University, Ganzhou, Jiangxi, China; ^3^ Key Laboratory of Urology and Andrology of Ganzhou, Ganzhou, Jiangxi, China; ^4^ Department of Graduate, The First Affiliated Hospital of Gannan Medical University, Ganzhou, Jiangxi, China

**Keywords:** urologic oncologies, lncRNA, autophagy, pathways, molecular mechanisms

## Abstract

Urologic oncology is a significant public health concern on a global scale. Recent research indicates that long chain non-coding RNAs (lncRNAs) and autophagy play crucial roles in various cancers, including urologic malignancies. This article provides a summary of the latest research findings, suggesting that lncRNA-mediated autophagy could either suppress or promote tumors in prostate, kidney, and bladder cancers. The intricate network involving different lncRNAs, target genes, and mediated signaling pathways plays a crucial role in urological malignancies by modulating the autophagic process. Dysregulated expression of lncRNAs can disrupt autophagy, leading to tumorigenesis, progression, and enhanced resistance to therapy. Consequently, targeting particular lncRNAs that control autophagy could serve as a dependable diagnostic tool and a promising prognostic biomarker in urologic oncology, while also holding potential as an effective therapeutic approach.

## Introduction

Urologic tumors encompass a significant array of diseases such as prostate, kidney, bladder, adrenal, ureteral, and testicular tumors. Prostate, bladder, and kidney cancers are the most prevalent urological cancers worldwide, with approximately 2.4 million new cases reported annually, representing a substantial portion of male cancer diagnoses and contributing to 10% of cancer-related deaths (Sung et al., 2021). In 2019, global fatalities attributed to kidney, bladder, and prostate cancers totaled 1,66,440, 2,28,730, and 4,86,840 cases, respectively (Tian et al., 2023). As the population ages, the incidence of urologic cancers is expected to rise significantly, potentially imposing a substantial burden on the global economy. It is estimated that the annual cost of treating prostate, kidney, and bladder cancers in the United States in 2020 will reach $31.47 billion (Mariotto et al., 2011). Thus, there is a pressing need to prioritize screening and intervention for urologic cancers to address the potential economic challenges that may arise in the future.

Despite advancements in diagnosing and treating urologic tumors, they continue to be the primary cause of death among urologic diseases, particularly in patients with advanced or metastatic disease. Radical surgery remains the mainstay treatment for early-stage urologic cancers. However, in cases of advanced tumors, treatment efficacy is often limited by significant resistance to chemotherapy or radiotherapy, resulting in a poorer prognosis.

An in-depth investigation of the molecular mechanisms underlying the pathogenesis of urologic tumors is crucial for advancing the diagnosis, treatment, and prognosis of these diseases. A pressing concern is enhancing the sensitivity of urologic cancers to radiotherapy and chemotherapy. Autophagy is widely recognized to play a significant role in the progression of various cancers, including urologic cancers (Yang et al., 2011). In diagnosed cancers, autophagy can participate in the reprogramming of the cellular microenvironment and protect cancer cells from different survival stresses (e.g., hypoxia, nutritional deficiencies, or cancer treatments) (Folkerts et al., 2019), thus favoring tumor progression. An expanding body of research has demonstrated that long chain non-coding RNAs (lncRNAs) play a role in promoting apoptosis through the regulation of autophagy (Ghafouri-Fard et al., 2022a). Most long non-coding RNAs (lncRNAs) primarily act as sponges, sequestering autophagy-related microRNAs (miRNAs) away from their intended targets (Tay et al., 2014; Yoon et al., 2014). In addition to this role, lncRNAs also play more intricate roles in regulating autophagy, such as chromatin and histone remodeling, transcriptional regulation, and protein-protein interactions (Ernst and Morton, 2013). Nevertheless, the precise mechanisms by which lncRNAs impact the progression of urinary tumors through autophagy regulation are not yet fully understood and lack comprehensive development. This review aims to comprehensively analyze existing literature to elucidate the significance of the lncRNA-autophagy axis in the development and prognosis of human urological oncology, offering insights for potential therapeutic interventions.

### Overview of autophagy

Cellular autophagy, also known as type II programmed cell death, is the self-digestive process in which cells use lysosomes to break down damaged, denatured, or senescent macromolecules and organelles in response to external environmental factors. This self-protection mechanism is common in eukaryotic cells and is crucial for regulating cell survival and death (Singh and Cuervo, 2011). In current research on autophagy function, the physiological roles of cellular autophagy are threefold. Firstly, it serves as a source of intracellular energy, crucial for maintaining energy metabolism and responding to stressful changes. Secondly, autophagy is vital in preserving cellular homeostasis. Lastly, programmed cell death triggered by autophagy is essential for eliminating unwanted or pathologically altered cells from the body. While autophagy is acknowledged as a mechanism for cellular self-preservation, excessive upregulation of autophagy-related genes can lead to cell death (Cheng et al., 2013). Autophagy can be induced in cells under various conditions such as starvation, endoplasmic reticulum stress, hypoxia, and radiation. This process is regulated by autophagy-related genes, leading the cell to encapsulate cytoplasm or organelles in vesicle-like autophagosomes through monolayer or bilayer membranes. Subsequently, the autophagosome merges with the lysosome to form an autolysosome, where hydrolytic enzymes break down the contents, enabling cell metabolism and energy renewal (Mizushima, 2007). Research has demonstrated that dysfunction in autophagy can contribute to the onset of significant diseases including tumors, cardiovascular diseases, neurodegenerative diseases, and immune disorders (Gatica et al., 2015; Mizushima, 2018; Yang and Klionsky, 2020). Consequently, investigating the occurrence, progression, molecular mechanisms, and regulation of autophagy holds substantial importance.

### The role of autophagy in urological cancers

With the advancement of autophagy research, numerous studies have revealed a significant correlation between cellular autophagy and tumors. In 1999, Levine et al. identified the tumor suppressor BECN1, marking the initial elucidation of the link between autophagy and tumors (Liang et al., 1999). The relationship between cellular autophagy and tumors is intricate, playing a dual role in tumor development. In the early stages of tumorigenesis, inhibiting cellular autophagy can enhance tumor cell growth, indicating its potential to suppress tumorigenesis. However, as tumors progress, cellular autophagy can impede apoptosis and facilitate tumor cell metastasis, leading to the sustained proliferation of tumor cells (White and DiPaola, 2009). In the early stages of tumorigenesis, autophagy can function as a tumor suppressor by breaking down and recycling damaged proteins and organelles to prevent their buildup. This includes inhibiting the accumulation of carcinogenic p62 protein aggregates and safeguarding against chronic tissue damage, inflammation, and genomic instability, ultimately reducing the risk of tumor initiation, growth, spread, and metastasis (White, 2012; Guo et al., 2013; Barnard et al., 2016). In the middle or late stages of tumor progression, autophagy can contribute to cell protection, serve as a defense mechanism, and promote survival by decreasing DNA damage, preserving mitochondrial function, and bolstering cancer cell resistance to stress. This ultimately sustains tumor metabolism, growth, and progression (White, 2012; Barnard et al., 2016). Furthermore, autophagy can also stimulate tumor cell migration by promoting blood vessel formation, enhancing tumor cell invasiveness, and enabling apoptotic evasion following radiotherapy and pharmacological chemotherapy (Fulda, 2018).

The role of cellular autophagy in tumor progression is bidirectional. Studies have shown that autophagy can act as an oncogenic factor in prostate, kidney, and bladder cancers, while also inhibiting tumor progression (Naponelli et al., 2015; Li et al., 2019; Choi, 2020). Additionally, autophagy has been found to promote the progression of urinary tract tumors through various mechanisms. In mammalian cells, autophagy involves a series of autophagy-associated proteins (ATGs) across six stages: initiation, nucleation, extension, closure, maturation, and degradation. This process is characterized by the fusion of autophagosomes and lysosomes to form autophagic lysosomes, leading to the bulk degradation of long-lived proteins and organelles. ATG5, a crucial component of autophagosome formation, plays a key role in the initiation, nucleation, extension, and closure stages by sequestering cytoplasmic material prior to its delivery to lysosomes (Ariosa and Klionsky, 2016). Conversely, autophagy has also been found to promote the progression of urinary system cancer. ATG5, a crucial component in the formation of autophagosomes, plays a key role in sequestering cytoplasmic material for subsequent delivery to lysosomes. Research has shown that ATG5 expression is altered in prostate cancer cells, with upregulation particularly in the cytoplasm compared to normal prostate cells (Kim et al., 2011; Li et al., 2015). Patergnani et al. discovered that autophagy enhances cell proliferation and migration in renal cancer cells by degrading p53. They observed that inhibiting autophagy activation led to reduced p53 degradation, effectively suppressing the proliferative and migratory capabilities of renal cancer cells (Patergnani et al., 2020). Zhu et al. demonstrated that the autophagy-related gene Atg7 is notably upregulated in invasive bladder cancer. Furthermore, their findings showed that suppressing Atg7 expression led to a substantial decrease in bladder cancer invasion. These results indicate that Atg7 is involved in the regulation of bladder cancer cell invasion and that autophagy facilitates bladder cancer invasion (Zhu et al., 2019). Overall, these studies imply a close relationship between autophagy and the development of urological tumors.

There are currently five major pathways known to be involved in autophagy, which have been extensively researched. PTEN promotes autophagy by inhibiting the PI3K/AKT pathway, while AMPK enhances autophagy by inhibiting the AKT/mTOR pathway. This ultimately leads to the inhibition of the PI3K/AKT/mTOR signaling pathway that is activated in nutrient-rich environments. mTOR inhibits autophagy by acting through P70S6K and activator of transcription (STAT) 3 expression. The Wnt/β-catenin signaling pathway inhibits Beclin1-mediated autophagy, while the EGFR/Ras/MEK/ERK pathway and p38MAPK signaling pathway also inhibit autophagy. On the other hand, the activation of JNK/cJun signaling pathway promotes autophagy. It is worth noting that the PI3K/AKT/mTOR signaling pathway is particularly intriguing in the context of autophagy regulation (Wang et al., 2021).

In addition to various autophagy-associated pathways and proteins, autophagy also closely interacts with lncRNAs and lncRNA-triggered signaling. Numerous studies have demonstrated the involvement of a significant number of lncRNAs in the regulation of autophagy.

### LncRNAs

Non-coding RNAs are transcripts that do not encode proteins, yet they represent a substantial portion of the genome, particularly lncRNAs. Generally, lncRNAs are transcripts that exceed 200 nucleotides in length. This category encompasses enhancer lncRNAs, intron/intergenic lncRNAs, and positive/antisense lncRNAs, with diverse isoforms contributing to a complex and heterogeneous group (Rinn and Chang, 2012; Boon et al., 2016). LncRNAs have diverse functions in genetic information transmission, including chromatin reprogramming, transcriptional regulation (e.g., cis-regulation of enhancers), post-transcriptional regulation (e.g., mRNA processing), and translational regulation. This has led to an increased interest in studying lncRNAs as they play a crucial role in complex biological processes and offer potential new therapeutic targets (Martianov et al., 2007; Tripathi et al., 2010; Prensner and Chinnaiyan, 2011; Ulitsky and Bartel, 2013; Stojic et al., 2016). Long non-coding RNAs (lncRNAs) play various key roles in biological processes, such as interfering with the transcription of nearby genes, regulating RNA polymerase II, mediating histone modifications, serving as precursors of microRNAs or siRNAs, modulating protein activity, and functioning as endogenous RNAs (ceRNAs) that competitively regulate miRNAs at the post-transcriptional level. This diverse range of functions allows lncRNAs to participate in a wide array of biological processes through the ceRNA/miRNA regulatory network (Zhong et al., 2022; Han et al., 2023). LncRNAs are closely associated with a variety of physio-pathological processes, including cardiovascular diseases, diabetes, Alzheimer’s disease, Parkinson’s disease, leukemia, tumors, and more (Ghafouri-Fard et al., 2022a).

The characteristics of lncRNAs indicate a potential link to the development and advancement of malignant tumors. Studies have demonstrated that lncRNAs can modulate gene expression, impacting cell growth, division, differentiation, and cell death, potentially leading to oncogenic effects. Additionally, certain lncRNAs are influenced by cancer transforming inhibitors (TIs) or oncogene products, indirectly contributing to oncogenic processes (Huarte, 2015; Schmitt and Chang, 2016). Cancer stem cells (CSC) are a specific group of cancer cells that possess the capacity to invade, spread, and metastasize. Numerous Long non-coding RNAs (LncRNAs) have been identified as regulators of stem cell function, influencing key transcription factors that maintain stemness, traditional pathways associated with stem cells, and related microRNAs. These regulatory mechanisms play a critical role in shaping the biology of CSCs, ultimately impacting cancer progression, prognosis, and the likelihood of recurrence (Schwerdtfeger et al., 2021). During tumor progression and treatment, cellular stress responses can regulate the fate of tumor cells and influence the sensitivity of therapeutic agents. LncRNAs play a role in regulating tumor progression and response to cancer treatment by being involved in molecular mechanisms related to oxidative, metabolic, hypoxic, genotoxic, and endoplasmic reticulum stress (Wu et al., 2022). Long non-coding RNAs (lncRNAs) have been documented to play a role in controlling cancer cell stemness and epithelial-mesenchymal transition (EMT), ultimately impacting cancer advancement and resistance to chemotherapy (McCabe and Rasmussen, 2021). These lncRNAs are intricately linked to the modulation of crucial signaling pathways like p53, AKT, and Notch, thereby exerting influence on cancer development (Peng et al., 2017). Furthermore, the metabolic reprogramming characteristic of cancer necessitates the participation of lncRNAs (Lin et al., 2020).

Recent studies have shown that lncRNAs play a role in mediating the autophagy process by regulating the expression of autophagy genes. These lncRNAs have been found to impact various cancer phenotypes, including survival, proliferation, epithelial-mesenchymal transition, migration, invasion, angiogenesis, and metastasis, by inhibiting microRNAs linked to autophagy. Furthermore, lncRNAs have been identified as regulators of autophagy in different types of cancer (Kumar et al., 2023). Key cellular signaling pathways involved in cell survival and metabolism, such as Akt (protein kinase B, PKB), mammalian target of rapamycin (mTOR), and AMPK, regulate autophagy. The expression of key members of upstream cellular signaling, as well as those directly involved in the mechanisms of autophagy and apoptosis, is controlled by microRNAs (miRNAs) and lncRNAs. There is growing evidence that various lncRNAs regulate autophagy in different types of cancer. Various lncRNAs targeting the autophagic process have been described in cancers such as bladder, breast, cervical colon, lung, liver, blood, bone, brain, pancreatic and prostate (Kumar et al., 2023). Therefore, lncRNAs play a significant role in cancer carcinogenesis and therapeutic response through the regulation of autophagy (de la Cruz-Ojeda et al., 2022). The aforementioned results led us to conduct a thorough examination of the existing literature to understand how lncRNAs regulate autophagy in relation to urologic cancers. This investigation may lead to the development of novel therapeutic approaches for targeting urologic cancers more effectively.

### Regulation of autophagy in urologic cancers by lncRNAs

(Table 1).

**TABLE 1:** The role of autophagy modulated by LncRNAs in cancer initiation and cancer development.

Cancers	Vitro/vivo experiments	Experimental methods	LncRNA	Target gene/Regulatory pathway	Pro/Antitumor	Anti-/Proautophagy	References
Prostate cancer	Vitro	RNA-seq,smRNA-seq,RT-qPCR,IHC	LINC01801	has-miR-6889-3p	Pro-tumor	Proautophagy	Chang et al. (2023)
	Vitro	RT-qPCR,WB,ETM	RHPN1-AS1	miR-7-5p/EGFR	Pro-tumor	Antiautophagy	Ma et al. (2022)
	Vitro	MS, RNA pull-down,RT-qPCR,WB,ETM,RIP	LncRNA PCDRlnc1	UHRF1	Pro-tumor	Proautophagy	Xie et al. (2022)
	Vitro and vivo	RNA pull-down,RT-qPCR,WB,ETM	LncAY927529	CXCL14	Pro-tumor	Proautophagy	Li et al. (2021)
	Vitro and vivo	RT-qPCR,WB,MTT, Transwell,FCM	LncRNA SNHG1	EZH2	Pro-tumor	Antiautophagy	Chen et al. (2020)
	Vitro	RT-qPCR,IHC	LncRNA PCGEM1	Not mentioned	Pro-tumor	Antiautophagy	Han et al. (2020)
	Vitro and vivo	RT-qPCR,WB,ETM,PI, Scratch test	LncRNA PRRT3-AS1	PPARγ/mTOR	Pro-tumor	Antiautophagy	Fan et al. (2020a)
	Vitro and vivo	RT-qPCR,IHC,RIP,WB, ChIP	LncRNA IDH1-AS1	Not mentioned	Pro-tumor	Proautophagy	Zhang et al. (2019)
	Vitro and vivo	RT-qPCR,WB, RNA pull-down	LncRNA HULC	mTOR	Pro-tumor	Antiautophagy	Chen et al. (2018)
	Vitro	RT-qPCR, qPCR, RNA-seq	LINC01116	Not mentioned	Pro-tumor	Antiautophagy	Beaver et al. (2017)
Bladder cancer	Vitro and vivo	RT-qPCR,WB,IHC	LncRNA MEG3	miR-93-5p	Antitumor	Antiautophagy	Fan et al. (2020b)
	Vitro and vivo	qPCR,WB,Transwell,FCM	LncRNA TUG1	miR-582-5p	Pro-tumor	Proautophagy	Lu et al. (2023)
	Vitro and vivo	RT-qPCR,WB,IP	LncRNA H19	Not mentioned	Pro-tumor	Proautophagy	Guo et al. (2022)
	Vitro and vivo	WB, Transwell,IHC	LncRNA UCA1	miR-582-5p	Pro-tumor	Proautophagy	Wu et al. (2019)
	Vitro	RT-qPCR,WB,FCM, Transwell	ADAMTS9-AS1	PI3K/AKT/mTOR	Pro-tumor	Antiautophagy	Yang et al. (2021a)
	Vitro	qPCR,WB	ADAMTS9-AS2	PI3K/AKT/mTOR	Antitumor	Proautophagy	Zhang et al. (2020)
Kidney cancer	Vitro and vivo	RIP, RNA pull-down	LncRNA IGFL2-AS1	TP53INP2	Pro-tumor	Proautophagy	Pan et al. (2023)
	Vitro and vivo	IHC, RT-qPCR,WB, FISH, RIP, RNA pull-down, Transwell	LncRNA SBF2-AS1	miR-338-3p/ETS1	Pro-tumor	Antiautophagy	Yang et al. (2021b)
	Vitro and vivo	RT-qPCR,WB, RIP, RNA pull-down	LncRNA TUG1	miR-31-5p	Pro-tumor	Antiautophagy	Lv et al. (2020)
	Vitro and vivo	RT-qPCR,WB,EdU, Transwell,ETM,IHC	LncRNA HOTTIP	PI3K/Akt/Atg13	Pro-tumor	Antiautophagy	Su et al. (2019)
	Vitro	RT-qPCR,WB,MTT	LncRNA KIF9-AS1	miR-497-5p/TGF-β	Pro-tumor	Proautophagy	Jin et al. (2020)
	Vitro and vivo	RT-qPCR,WB,MTT, bioinformatic	LncRNA SCAMP1	miR-429/ZEB1/JUN	Pro-tumor	Antiautophagy	Shao et al. (2019)

RT-qPCR, Real-time reverse transcription and quantitative PCR; RNA-seq and smRNA-seq, High-throughput RNA and small RNA sequencing; IHC, Immunohistochemistry; WB, Western blot analysis; ETM, Transmission electron microscopy; RIP, RNA immunoprecipitation; MS, mass spectrometry; FCM, flow cytometry; PI, Propidium iodide; ChIP, Chromatin immunoprecipitation; IP, Immunoprecipitation; FISH, Fluorescence *in situ* hybridization; EdU, Ethynyldeoxyuridine.

### Interaction of lncRNA and autophagy in PCa

#### LINC01801/has-miR-6889-3p signaling

**FIGURE 1:**
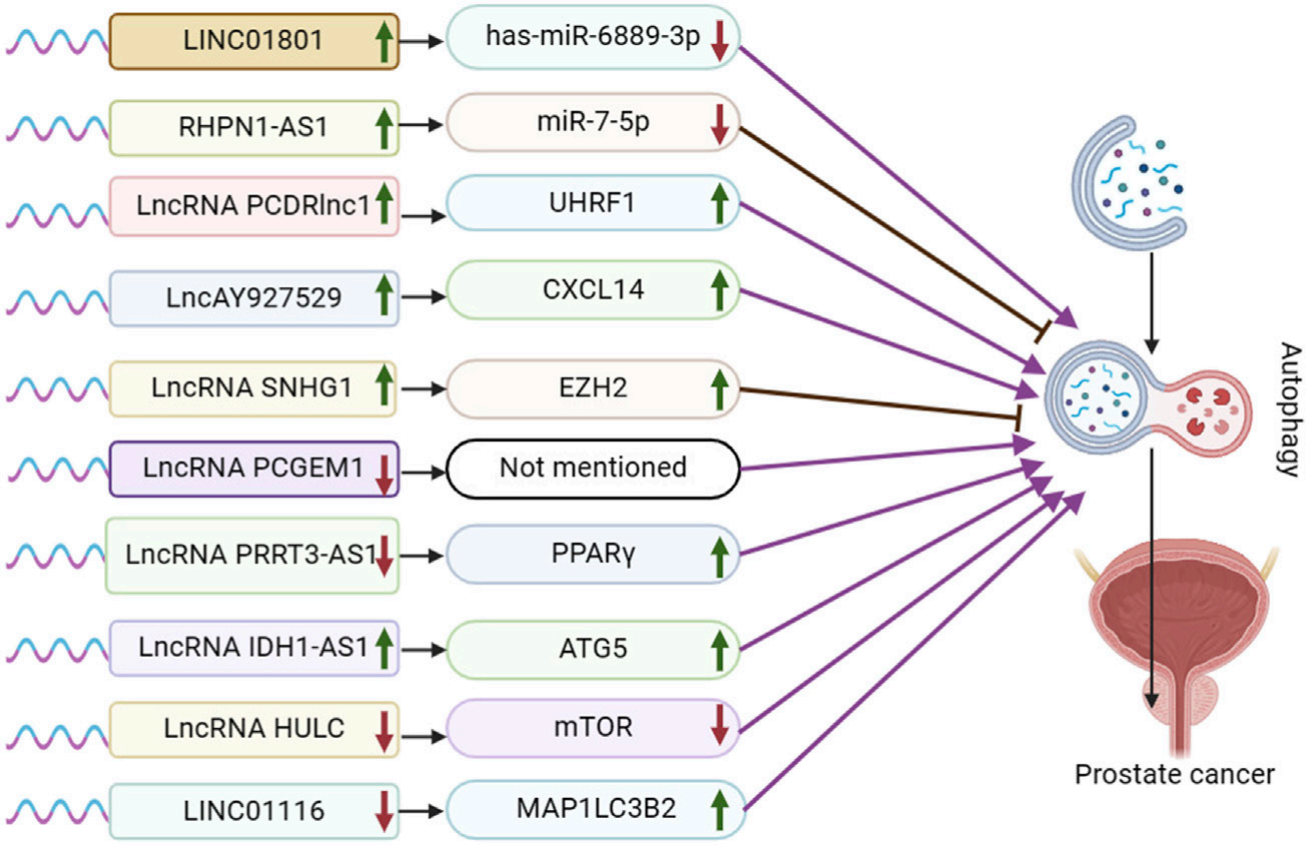
Schematic representation of the association between LncRNA-mediated autophagy and prostate cancer. UHRF1:Ubiquitin-like with PHD and ring finger domains 1; CXCL14: the chemokine CXCL14; EZH2: Enhancer of zeste homolog 2; PPARγ: peroxisome proliferator-activated receptor γ; ATG5: autophagy-related protein ATG5. ATG5, mTOR, and MAP1LC3B2 are autophagy pathway-related proteins, not target genes or regulatory pathways of LncRNA IDH1-AS1 and LINCO1116.

In the lncRNA and autophagy literature, prostate cancer is the most extensively researched urological tumor. Following standard androgen deprivation therapy, prostate cancer can advance to castration-resistant prostate cancer (CRPC). Subsequent treatment with next-generation AR-targeted therapies like abiraterone and enzalutamide may lead to resistance, metastasis, and progression to neuroendocrine prostate cancer (NEPC) in some patients (Puca et al., 2019). The repressor-1 silencing transcription factor (REST) protein is known for its role as a transcriptional repressor that silences neuronal genes in non-neuronal cells and maintains pluripotency in neural precursor cells (Ooi and Wood, 2007). REST has been shown to regulate lncRNA HOTAIR and facilitate neuroendocrine differentiation in castration-resistant prostate cancer (Chang et al., 2018). In the disquisition by Chang et al., it was found that LINC01801 was shown to activate autophagy during NED by sponging miR-6889-3p and up-regulating the transcription of autophagy-related genes (ATM, HIF1A, PTEN, TBK1, VPS13A, and XPO1). REST represses the lncRNA LINC01801, which induces neuroendocrine differentiation in prostate cancer by mediating the transcription of autophagy-related genes and activating autophagy when REST is reduced (Chang et al., 2023) (Figure 1).

#### LncRNA RHPN1-AS1/miR-7-5p/EGFR/PI3K/AKT/mTOR signaling

The lncRNA RHPN1 antisense RNA 1 (RHPN1-AS1) has been identified as being dysregulated in various cancers, such as breast cancer, squamous cell carcinoma, and non-small cell lung cancer, across multiple research studies (Yu et al., 2023). Ma et al. demonstrated that RHPN1-AS1 is upregulated in both prostate cancer (PCa) tissues and cells, leading to reduced survival time in PCa patients. Silencing RHPN1-AS1 resulted in decreased proliferation and invasion of PCa cells, along with G2/M blockade, promoting apoptosis and autophagy. By binding to miR-7-5p, RHPN1-AS1 inhibits its expression, subsequently impacting PCa cells. Conversely, miR-7-5p inhibits PC3 cell proliferation, enhances autophagy and apoptosis, and is implicated in PCa development. Furthermore, RHPN1-AS1 overexpression upregulates epidermal growth factor receptor (EGFR) expression, while miR-7-5p overexpression counteracts this effect. Through this interaction, RHPN1-AS1 acts as a competitive endogenous RNA (ceRNA) for miR-7-5p, ultimately elevating EGFR expression. The PI3K/AKT-mTOR pathway downstream of EGFR is crucial in regulating autophagy progression, with RHPN1-AS1 overexpression activating this pathway. Additionally, RHPN1-AS1 overexpression decreased protein-cleaving cysteine-3 expression, involved in apoptosis, and reduced apoptotic cell numbers in PC3 and LNCaP cells. In summary, RHPN1-AS1 functions as a ceRNA for miR-7-5p, upregulating EGFR expression and inducing autophagy and apoptosis in PCa cells via the PI3K/AKT/mTOR pathway (Ma et al., 2022).

#### PCDRlnc1/UHRF1/Beclin-1 signaling

In advanced stages of prostate cancer, docetaxel is commonly used as the initial standard treatment. However, drug resistance can reduce its effectiveness over time (Heidenreich et al., 2014). Paclitaxel inhibits autophagy through two distinct mechanisms dependent on cell cycle phase. In mitotic cells, paclitaxel blocked the activation of class III phosphatidylinositol 3 kinase (Vps34), a key initiator of autophagosome formation. In non-mitotic paclitaxel-treated cells, autophagosomes were produced, but autophagosome motility was inhibited, preventing autophagosome maturation (Veldhoen et al., 2013). Mitochondrial autophagy is believed to play a crucial role in the development of resistance in docetaxel-resistant prostate cancer (Cristofani et al., 2018). Moreover, the activation of signal transducer STAT3 inhibits docetaxel-induced autophagy, leading to the regulation of autophagy and the promotion of drug resistance in prostate cancer cells (Hu et al., 2018). Xie et al.'s experimental result demonstrated that PCDRlnc1 plays a role in promoting autophagy and docetaxel resistance in PCa. They observed a significant decrease in the expression of Beclin-1, a key autophagy regulator, when PCDRlnc1 was silenced, whereas overexpression of PCDRlnc1 led to an increase in Beclin-1 levels. Furthermore, the levels of UHRF1 mRNA and protein were found to be positively associated with PCDRlnc1 expression. The disquisition concluded that PCDRlnc1 facilitates autophagy and docetaxel resistance in PCa by modulating UHRF1-induced Beclin-1 expression (Xie et al., 2022).

#### LncAY927529/CXCL14 signaling

Exosomes are small membrane vesicles with a lipid bilayer structure that are secreted by most cells, typically measuring about 30–200 nm in diameter (Pegtel and Gould, 2019). They can be found in body fluids and have the ability to travel through the extracellular matrix, facilitating communication between cells by merging with the cell membrane of neighboring cells (Meldolesi, 2018). Exosomes are involved in various physiological and pathological processes, including tumor cell proliferation and metastasis (Whiteside, 2016). Meldolesi’s study revealed that LncAY927529 levels were elevated in the serum of prostate cancer patients as well as in exosomes of prostate cancer cells. Additionally, LncAY927529 was found to enhance the proliferation and invasion of prostate cancer cells. CXCL14, a CXC chemokine, is a small secreted protein that facilitates the directional migration of cells and plays a crucial role in various physiological and pathological processes. Meldolesi et al. validated lncAY927529 and CXCL14 through RPIseq and Pull down assay prediction. They discovered that CXCL14 acts as an RNA-binding protein for lncAY927529, and that exosome-mediated lncAY927529 regulates CXCL14 levels in ST2 cells. Ultimately, lncAY927529 promotes a conducive microenvironment for tumor growth by facilitating autophagy induction through CXCL14 (Li et al., 2021).

#### SNHG1/EZH2/PI3K/AKT/mTOR/Wnt/β-catenin signaling

Various studies have demonstrated that the long non-coding RNA nucleolar small molecule RNA host gene 1 (SNHG1), located on chromosome 11, is upregulated as an oncogene in multiple tumor types (including lung, liver, gastric, colorectal, esophageal, and prostate cancer) and is closely linked to a poor prognosis in these malignancies (Zeng et al., 2023). The Polycomb Repressive Complex 2 (PRC2) is a highly conserved histone methyltransferase, with EZH2 being the catalytic subunit and a central component of the PRC2 protein complex (Jiang et al., 2016). Chen et al. demonstrated that mRNA expression of SNHG1 and EZH2 was significantly higher in PCa tissues compared to adjacent normal tissues. SNHG1 was found to enhance EZH2 expression, thereby influencing PCa cell proliferation, hyperproliferation, migration, and invasion by targeting EZH2. Moreover, SNHG1 binding to EZH2 was shown to suppress cellular autophagy through activation of the PI3K/AKT/mTOR and Wnt/β-catenin pathways, ultimately promoting prostate cancer cell proliferation (Chen et al., 2020).

#### PCGEM1/LC3-II signaling

Prostate Cancer Gene Expression Marker 1 (PCGEM1) has been identified and patented as a regulator of prostate cancer progression (Ifere and Ananaba, 2009). Moreover, research has demonstrated a strong association between PCGEM1 and the advancement of cervical, endometrial, gastric, ovarian, hepatocellular, and renal cancers (Ghafouri-Fard et al., 2022b). Baicalein, a monomer extracted from Scutellaria baicalensis, has been linked to tumor cell proliferation, invasion, and metastasis in adenocarcinoma, bladder cancer, breast cancer, and various other cell lines (Morshed et al., 2023). In an academic study conducted by Han et al., it was found that LV3-shRNAPCGEM1 in combination with baicalein downregulated the expression of PCGEM1 and induced autophagy in LNCaP cells. Additionally, an increase in LC3-II expression was observed in the LNCaP cell line, indicating that reduced PCGEM1 expression could potentially trigger autophagy in prostate cancer cells (Han et al., 2020).

#### LncRNA PRRT3-AS1/PPARγ/mTOR signaling

In a research by Zhou et al., it was found that PRRT3-AS1 exhibited oncogenic effects on non-small cell lung cancer (Zhou et al., 2021). Peroxisome proliferator-activated receptors (PPAR) are ligand-activated receptors belonging to the nuclear hormone receptor family. They play a crucial role in regulating various intracellular metabolic processes, including anti-inflammatory and antioxidant effects. In the academic study by Li et al., it was found that lncRNA PRRT3-AS1 targeted the gene PPARγ. The expression of lncRNA PRRT3-AS1 was notably high in prostate cancer cells, while PPARγ expression was low. Silencing lncRNA PRRT3-AS1 or overexpressing PPARγ has been shown to impact cell cycle distribution, promoting apoptosis in PC cells. Additionally, this intervention promotes autophagy, inhibits proliferation, growth, migration, and invasion of prostate cancer cells by suppressing the activation of the mTOR signaling pathway (Fan et al., 2020).

#### LncRNA IDH1-AS1/ATG5 signaling

Isocitrate dehydrogenase (IDH) 1 and 2 are enzymes that function in the cytoplasm and mitochondria, respectively. They are NADPH-dependent enzymes involved in metabolizing isocitrate to α-ketoglutarate (α-KG) within the tricarboxylic acid cycle. Xiang et al. suggest that IDH1 plays a role in tumor cell growth, proliferation, and survival by regulating the levels of reactive oxygen species (ROS). Additionally, the IDH1-AS1-IDH1 axis may exhibit tumor suppressor properties (Xiang et al., 2018). Zhang et al. identified PAX5 as a potential upstream transcriptional regulator of IDH1-AS1 by screening the UCSC online database. Both PAX5 and IDH1-AS1 showed higher expression levels in PCa tissues and cell lines compared to normal tissues. PAX5 was found to enhance the expression of IDH1-AS1, leading to increased growth of PCa cells both *in vitro* and *in vivo*. Additionally, IDH1-AS1 was observed to interact with PTBP3 to regulate ATG5 mRNA stability. Furthermore, IDH1-AS1 promotes ATG5-mediated PCa autophagy by sponging miR-216b-5p to upregulate ATG5 expression (Zhang et al., 2019).

#### LncRNA HULC/mTOR/Beclin-1 signaling

Hepatocellular carcinoma highly upregulated (HULC) was first discovered during the screening of hepatocellular carcinoma (HCC)-specific gene libraries. Numerous studies have since confirmed that HULC serves as a new biomarker for carcinogenesis across various types of cancer (Chen et al., 2017; Ghafouri-Fard et al., 2020a). Chen et al. found that knocking down HULC effectively enhanced apoptosis and proliferation alterations induced by irradiation, while also decreasing the sensitivity of PC3 and LNCaP cells to irradiation. HULC inhibits Beclin-1 phosphorylation through regulation of the mTOR pathway. Knockdown of HULC results in elevated Beclin-1 phosphorylation and suppression of the mTOR pathway, ultimately triggering autophagy (Chen et al., 2018).

#### LINC01116/MAP1LC3B2 signaling

The long intergenic non-protein coding RNA 1116 (LINC01116) is located in the 2q31.1 region and has been identified as a key regulator of cancer cell proliferation, migration, and invasion. LINC01116 is closely linked to various types of tumors, such as lung cancer (LC), gastric cancer (GC), breast cancer (BC), colorectal cancer (CRC), glioma, and osteosarcoma (Xu et al., 2021). In the presence of MAPK/JNK or MTOR inhibitors, the autophagy pathway protein MAP1LC3B2/LC3B-II is upregulated, leading to organelle autophagy loss and the conversion of MAP1LC3B1 to MAP1LC3B2, which is essential for autophagosome formation (Basu et al., 2014; Ahsan et al., 2024). In a report by Laura M. Beaver et al. it was found that knockdown of LINC01116 induced autophagy by upregulating MAP1LC3B2 gene expression in prostate cancer cells (Beaver et al., 2017).

### Interaction of autophagy and lncRNA in bladder cancer

#### MEG3/miR-93-5p/PI3K/AKT/mTOR signaling

**FIGURE 2:**
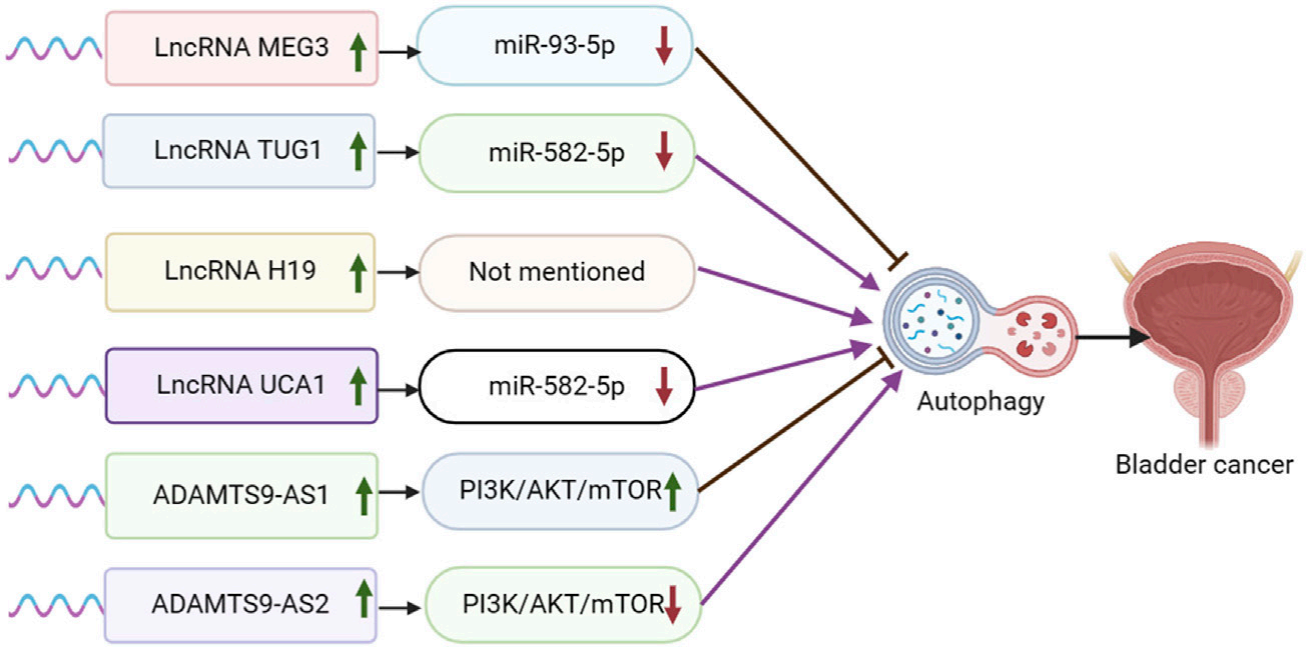
Schematic representation of the association between LncRNA-mediated autophagy and bladder cancer.

Maternal expressed gene 3 (MEG3) is a lncRNA that regulates target genes through transcription, translation, post-translational modification, and epigenetic regulation. MEG3 functions by sponging miRNAs, inducing cell death, regulating various cancer-related processes, inhibiting tumor cell proliferation, negatively regulating tumor cell invasion and metastasis, inhibiting tumor angiogenesis, and acting as a tumor suppressor. It is closely associated with various types of cancer, such as breast cancer, ovarian cancer, colorectal cancer, lung cancer, and hepatocellular carcinoma (Zhang et al., 2022). Fan et al. discovered that MEG3 was downregulated and miR-93-5p was upregulated in bladder cancer cell lines and tissues. They also found that MiR-93-5p was a direct target of MEG3, which competitively inhibited miR-93-5p expression. Furthermore, MEG3 overexpression was shown to inhibit autophagy and activation of the PI3K/AKT/mTOR pathway by targeting miR-93-5p (Fan et al., 2020) (Figure 2).

#### TUG1/miR-582-5p/PI3K/AKT signaling

Long-chain non-coding RNA taurine upregulated gene 1 (TUG1) is implicated in the prognosis of several human malignant tumors such as hepatocellular carcinoma, colorectal carcinoma, breast cancer, cervical carcinoma, and gastric cancer (Wang et al., 2017). In bladder cancer (BC), high expression of TUG1 is linked to a poor prognosis (Jiang et al., 2023). Additionally, abnormal expression of miR-582-5p is associated with bone metastasis in prostate cancer (PCa) (Huang et al., 2019). Lu et al. demonstrated that TUG1 expression was elevated and miR-582-5p expression was reduced in drug-resistant bladder cancer tissues and cells. They found that TUG1 functioned as a sponge for miR-582-5p, leading to the inhibition of miR-582-5p expression. Additionally, TUG1 was shown to modulate the PI3K/AKT pathway by sponging miR-582-5p, consequently promoting autophagy and enhancing resistance of bladder cancer cells to DOX chemotherapy (Lu et al., 2023).

#### LncRNA H19/ULK1 signaling

Long non-coding RNA H19 (lncRNA H19) has been extensively linked to the progression and outcome of various tumors, notably in breast, colorectal, liver, and lung cancers. Moreover, lncRNA H19 plays a crucial role in modulating the effectiveness of chemotherapy, endocrine therapy, and targeted therapy in these cancer types (Zhang et al., 2022). Guo et al. discovered a notable increase in H19 expression in TAMs-Exo. The overexpression of H19 in TAMs-Exo led to a significant enhancement of LC3-II expression, thereby promoting autophagy in bladder cancer cells. The overexpression of lncRNA H19 stabilized ULK1 expression and facilitated autophagy in bladder cancer cells by disrupting the interaction between NEDD4L and ULK1 (Guo et al., 2022).

#### UCA1/miR-582-5p/ATG7 signaling

Urothelial carcinoma-associated 1 (UCA1) is a long non-coding RNA that is highly expressed in most tumor tissues, particularly in bladder cancer. It plays a crucial role in regulating various processes in tumor cells, such as proliferation, apoptosis, invasion, and migration (Li and Hu, 2015). Studies have demonstrated that UCA1 contributes to the progression of bladder cancer by increasing cell proliferation, invasion, and migration, while also decreasing apoptosis (Xue et al., 2014; Xue et al., 2016). In the report conducted by Wu et al., it was observed that UCA1 was highly expressed while miR-582-5p was lowly expressed in bladder cancer tissues. Furthermore, UCA1 was found to promote the growth, migration, and invasion of bladder cancer cells by directly inhibiting the expression of miR-582-5p. Additionally, miR-582-5p demonstrated the ability to directly inhibit the expression of ATG7. The knockdown of UCA1 resulted in the inhibition of cell growth and metastasis of bladder cancer cells by mediating the inhibition of autophagy through the miR-582-5p/ATG7 pathway. Therefore, UCA1 knockdown suppressed cell growth and metastasis of bladder cancer cells by inhibiting autophagy through the mediation of miR-582-5p/ATG7 (Wu et al., 2019).

#### ADAMTS9-AS1, AS2/PI3K/AKT/mTOR signaling

The lncRNA ADAMTS9-AS2 has been documented as being dysregulated in various types of tumors. It has been shown to play a role in cancer progression through different mechanisms, such as modulating miRNAs and activating traditional signaling pathways in cancer. These actions are associated with the suppression of cancer cell growth, invasion, migration, and programmed cell death (Xu et al., 2021). Zhang et al. discovered that the expression of ADAMTS9-AS2 was reduced in bladder tumor cells, leading to inhibition of proliferation, migration, and invasion in bladder cancer cells. Overexpression of ADAMTS9-AS2 in bladder cancer cells blocked the PI3K/AKT/mTOR signaling pathway, suggesting promotion of autophagy (Zhang et al., 2020). Conversely, ADAMTS9-AS1 exhibited contrasting results. Yang et al. reported that upregulation of ADAMTS9-AS1 enhanced proliferation, migration, and invasion in bladder cancer cells, while also suppressing apoptosis and autophagy (Yang et al., 2021).

### Interaction of autophagy and lncRNA in kidney cancer

#### LncRNA IGFL2-AS1/TP53INP2 signaling

**FIGURE 3:**
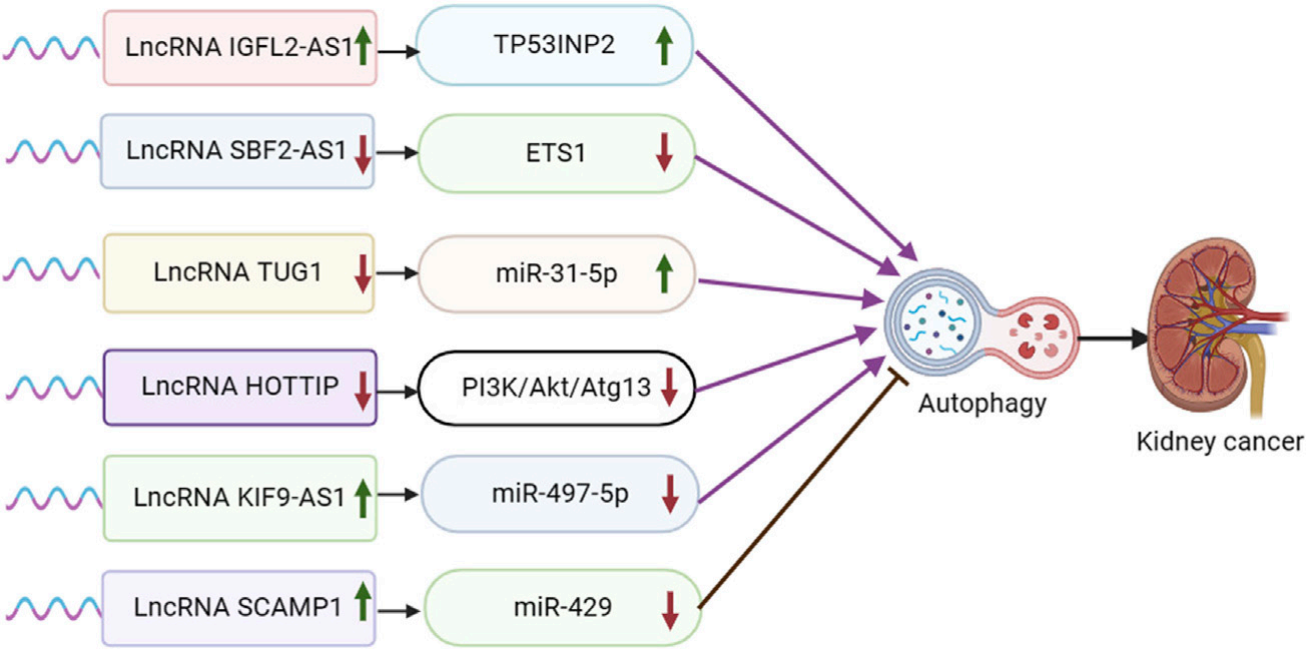
Schematic representation of the association between LncRNA-mediated autophagy and renal cancer. ETS1: ETS proto-oncogene 1.

Non-coding RNA IGFL2-AS1 has been shown to be upregulated in various tumor cell types, such as cervical, renal cell, gastric, and oral cancers. IGFL2-AS1 plays a role in regulating cell proliferation and metastasis by modulating Wnt/β-catenin signaling via SATB1 and AKT activation through serine/threonine phosphatase PP2A, among other mechanisms (Cheng et al., 2019; Ma et al., 2020; Bispo et al., 2021; Zhao et al., 2023). In a study conducted by Pan et al., it was discovered that IGFL2-AS1 is upregulated in clear cell renal cell carcinoma (ccRCC) patients who have undergone sunitinib treatment. Additionally, the transcriptional efficiency of TP53INP2 mRNA was significantly increased following sunitinib treatment. The experiment revealed that IGFL2-AS1 plays a role in regulating selective splicing and enhancing the expression of TP53INP2 by competitively binding with the RRM of hnRNPC, thereby promoting autophagy and contributing to sunitinib resistance (Pan et al., 2023) (Figure 3).

#### SBF2-AS1/miR-338-3p/ETS1 signaling

Long-stranded non-coding RNA SET binding factor 2 antisense RNA 1 (lncRNA SBF2-AS1) is an oncogenic antisense RNA located at the 11p15.1 locus. It is 2,708 nucleotides long and has been found to be variably expressed in various tumor tissues, such as breast cancer (BC), cervical cancer (CC), hepatocellular carcinoma (HCC), lung cancer (LC), and lung adenocarcinoma (LUAD) (Tan et al., 2022). Yang et al. discovered that SBF2-AS1 exhibited high expression levels in clear cell renal cell carcinoma (ccRCC) tissues and cells. They observed that SBF2-AS1 interacted with miR-338-3p, leading to its suppression, which in turn targeted ETS1. Upon silencing SBF2-AS1, there was a notable inhibition of tumor cell proliferation, migration, and invasion, while promoting autophagy and apoptosis. This suggests that the suppression of SBF2-AS1 hinders ccRCC progression by upregulating miR-338-3p and downregulating ETS1 (Yang et al., 2021).

#### TUG1/miR-31-5p/FLOT1 signaling

The lncRNA known as taurine upregulated gene 1 (TUG1) has been found to have strong connections with various types of tumors. It has been identified as a biomarker for esophageal squamous cell carcinoma, hepatocellular carcinoma associated with viral hepatitis C, lung carcinoma, bladder carcinoma, ovarian carcinoma, and osteosarcoma (Da et al., 2021). In the experimental result by Lv et al., it was found that TUG1 was significantly upregulated in ccRCC tissues and cells. Knockdown of TUG1 led to inhibited cell proliferation and induced apoptosis and autophagy in ccRCC cells. TUG1 was observed to directly act on and inhibit MiR-31-5p expression. Furthermore, FLOT1 was identified as a direct target of miR-31-5p and exhibited a negative interaction with it. Overexpression of FLOT1 counteracted the inhibitory effects of MiR-31-5p on cell proliferation and promoted apoptosis and autophagy. Overall, the experiment concluded that lncRNA TUG1 regulates FLOT1 expression by sponging miR-31-5p, thereby influencing cell proliferation, apoptosis, and autophagy in ccRCC (Lv et al., 2020).

#### HOTTIP/PI3K/Akt/Atg13 signaling

The distal HOXA transcript (HOTTIP) has been identified as an oncogenic lncRNA present in various types of cancer. It is suggested to have potential as both a biomarker and a therapeutic target for human malignancies (Ghafouri-Fard et al., 2020b). In a study conducted by Yang et al., high levels of HOTTIP expression were observed in renal cell carcinoma (RCC) tissues and cell lines. The experiment found that silencing HOTTIP led to the induction of autophagy through the PI3K/Akt/Atg13 signaling pathway, resulting in the inhibition of proliferation, migration, and invasion of RCC cells (Su et al., 2019).

#### KIF9-AS1/miR-497-5p/TGF-β signaling

Abnormal expression of lncRNA KIF9-AS1 has been reported in a variety of tumor cells and is closely associated with the progression of nasopharyngeal carcinoma (You et al., 2020), hepatocellular carcinoma (Yu et al., 2022), and ovarian cancer (Li and Zhan, 2020). In Jin et al. ’ s study, KIF9-AS1 was found to be highly expressed in renal cell carcinoma (RCC) tissue cells. The experimental results showed that overexpression of KIF9-AS1 in RCC cells significantly reduced the sensitivity of sorafenib treatment and promoted the progression of RCC by promoting drug resistance. The results showed that miR-497-5p was a potential effector of kif9-as1-enhanced chemoresistance in RCC, and TGF-β signaling was a key effector of the KIF9-AS1/miR-497-5p axis in RCC. Autophagy was involved in the chemoresistance of RCC regulated by KIF9-AS1/mir-497-5p.m.iR-497-5p directly targeted and regulated the autophagy-related gene ATG9A. LncRNA KIF9-AS1 regulates autophagy of RCC by mediating miR-497-5p and TGF-β, and enhances the chemoresistance of renal cell carcinoma to sorafenib (Jin et al., 2020).

#### SCAMP1/miR-429/ZEB1/JUN signaling

The LncRNA SCAMP1 has been reported to be associated with a variety of tumors, including osteosarcoma (Li et al., 2022), ovarian cancer (Song et al., 2020), and glioma (Zong et al., 2019). In an experimental study conducted by Shao et al., it was discovered that SCAMP1 levels were elevated in RCC cells and tumors, leading to an enhancement in RCC cell viability. The biological effects induced by SCAMP1 were found to be mediated by miR-429. This microRNA directly targeted the expression of ZEB1 and JUN, resulting in a downregulation of both proteins. Consequently, this downregulation reduced the proliferation and invasiveness of tumor cells. Furthermore, miR-429 was shown to inhibit autophagy by downregulating the expression of ZEB1 and SCAMP1. The experiment results highlighted the significant impact of autophagy on miR-429-regulated RCC tumorigenesis (Shao et al., 2019).

### Therapeutic potential related to lncRNA-autophagy mechanism

In the present report, it was found that lncRNA can influence the susceptibility of urologic cancer cells to radiotherapy and chemotherapy by regulating autophagy. Following radiotherapy or chemotherapy, patients with tumors frequently trigger autophagy in tumor cells. This process facilitates the recycling of energy by breaking down senescent and damaged organelles, providing energy for tumor cells undergoing apoptosis induced by radiotherapy or chemotherapy, ultimately leading to resistance to treatment (Liu et al., 2016; Li et al., 2019; Chen et al., 2021). Several studies have indicated that autophagy-associated lncRNAs (ARlncRNAs) play a role in mediating tumor cell resistance to radiotherapy and chemotherapy by impacting various cellular processes such as proliferation, apoptosis, migration, invasion, drug resistance, angiogenesis, radiation resistance, and immunomodulation in urological tumors (Zhang et al., 2024). For instance, Xie et al. found that the lncRNA PCDRlnc1 contributes to docetaxel resistance in prostate cancer by enhancing autophagy (Xie et al., 2022), while Wang et al. showed that the LncRNA FEZF1-AS1 promotes chemoresistance in prostate cancer through regulation of the miR-25-3p/ITGB8 axis (Wang et al., 2020). Chen et al. demonstrated in a prostate cancer study that overexpression of LncRNAHULC increased the resistance of DU-145 cells to irradiation by promoting autophagy (Chen et al., 2018). In a separate study on bladder cancer, Lu et al. found that lncRNA TUG1 reduced the sensitivity of bladder cancer cells to doxorubicin by upregulating KPNA2 expression and activating the PI3K/AKT autophagy pathway through sequestration of miR-582-5p (Lu et al., 2023). In the context of renal cancer cells, Jin et al. revealed that lncRNA KIF9-AS1 modulates transforming growth factor β and autophagy signaling via microRNA-497-5p, leading to enhanced resistance to chemotherapeutic agents like sorafenib in renal cell carcinoma (Jin et al., 2020). These findings offer novel insights into the involvement of lncRNA-mediated autophagy in urologic radiotherapy and chemotherapy. However, a prognostic model has identified a correlation between autophagy-associated long-stranded non-coding RNAs and the tumor microenvironment (TME), encompassing factors such as immunotherapy, cancer-associated inflammation, and metabolic reprogramming (Li et al., 2024). As a result, it is necessary to further investigate how the interaction of these lncRNA-mediated pathways with the autophagy pathway in cancer cells responds to chemotherapy and drug therapy in urologic tumors.

## Summary and prospect

This study represents the first systematic evaluation to summarize the existing evidence on the relationship between lncRNAs and autophagy in urologic oncology. The findings suggest that specific lncRNAs associated with urologic cancers may impact various aspects of cancer progression, such as cell proliferation, apoptosis, cell cycle regulation, migration, invasion, angiogenesis, and cellular senescence. This research highlights the dual role of lncRNA in regulating autophagy, with the ability to either activate or inhibit autophagy. Similarly, autophagy exhibits a dual regulatory effect on cancer progression, adding complexity to the understanding of how lncRNA influences cancer progression through autophagy. For instance, in bladder cancer, lncRNA TUG1, lncRNA H19, and lncRNA UCA1 have been shown to promote cancer progression by activating autophagy, while ADAMTS9-AS2 inhibits bladder cancer by activating autophagy. Conversely, lncRNA MEG3 inhibits bladder cancer progression by suppressing autophagy, and ADAMTS9-AS1 promotes bladder cancer progression by inhibiting autophagy. There are several limitations in this systematic evaluation. Firstly, the intricate nature of autophagy and its dual role in tumor progression make it challenging to fully understand its mechanism in urological tumors, necessitating further research. Secondly, aside from exploring the molecular mechanism of lncRNA-mediated autophagy in urological tumorigenesis, other factors such as tumor microenvironment and immunity require deeper investigation. Lastly, this academic research provides a systematic evaluation but lacks extensive research on clinical aspects like diagnosis, treatment, and prognosis. The role of autophagy in tumor progression is gaining recognition among researchers and is becoming more prevalent in the literature. Additionally, researchers are increasingly incorporating human specimens into their clinical studies, when ethical guidelines allow. It is important for future studies to delve deeper into the interplay between lncRNA-mediated autophagy and other mechanisms within the tumor environment that contribute to tumor progression. LncRNA-mediated autophagy is a significant factor in urologic tumorigenesis, progression, and resistance to anticancer therapy. This highlights the potential for targeting these lncRNAs to regulate autophagy as a reliable diagnostic and prognostic biomarker, as well as a promising therapeutic approach in urologic oncology. The review suggests that lncRNA-associated autophagy plays a crucial role in the advancement of urologic tumors, including prostate, bladder, and renal cancers. The regulation of lncRNAs can either promote or hinder autophagy within these cancer types. A network involving lncRNA-targeted genes and various signaling pathways intricately governs autophagy regulation, impacting the development and suppression of malignant tumors in the urinary system.
